# Insufficiency of quality of life as the treatment endpoint for balloon pulmonary angioplasty in inoperable chronic thromboembolic pulmonary hypertension

**DOI:** 10.2478/jtim-2022-0067

**Published:** 2024-05-21

**Authors:** Juanni Gong, Yuan Ding, Jianfeng Wang, Wei Wang, Qiang Huang, Ran Miao, Tuguang Kuang, Suqiao Yang, Jifeng Li, Xiaojing Jiao, Yuanhua Yang

**Affiliations:** Department of Respiratory and Critical Care Medicine, Beijing Institute of Respiratory Medicine and Beijing Chao-Yang Hospital, Capital Medical University, Beijing 100020, China; Department of Interventional Radiology, Beijing Chao-yang Hospital, Capital Medical University, Beijing 100020, China; Medical Research Center, Beijing Institute of Respiratory Medicine and Beijing Chao-Yang Hospital, Capital Medical University, Beijing 100020, China

**Keywords:** chronic thromboembolic pulmonary hypertension, inoperable, balloon pulmonary angioplasty, SF-36, quality of life

## Abstract

**Background and Objectives:**

The ability of a quality of life (QoL) to guide balloon pulmonary angioplasty (BPA) among patients with inoperable chronic thromboembolic pulmonary hypertension (CTEPH) has not been fully investigated. This study explored the relationship between QoL scores and hemodynamics in CTEPH patients after BPA and examined whether QoL could be applied as a treatment endpoint.

**Materials and Methods:**

This cohort study included patients with inoperable CTEPH who had undergone at least four sessions of BPA. The patients’ demographic and clinical data as well as hemodynamic parameters and scores from the RAND 36-item short-form QoL questionnaire were recorded and compared before and after BPA.

**Results:**

After BPA treatments, clinical characteristics, hemodynamic parameters, as well as QoL score improved significantly. A physical component summary (PCS) score of 35 or 46 can be used as the cutoff value for predicting better World Health Organization functional classification (WHO FC). Patients who had a higher PCS would have longer 6-min walk distance (6MWD), lower pulmonary vascular resistance (PVR), and better cardiac output (CO) both before and after BPA. However, 19 patients (55.9%) with a higher PCS score after BPA did not achieve the goal of mean pulmonary arterial pressure (mPAP) ≤30 mmHg. During the follow-up period, a significant reduction of PVR was observed, but the PCS score improved a little.

**Conclusions:**

QoL is a useful tool for assessing the exercise endurance of patients with inoperable CTEPH treated with BPA, but is insufficient to serve as a treatment endpoint for BPA.

## Introduction

Balloon pulmonary angioplasty (BPA) is an important treatment option for inoperable chronic thromboembolic pulmonary hypertension (CTEPH) that can improve the functional and hemodynamic status of patients after treatment.^[1, 2, 3, 4]^ However, the current assessment of the treatment effect of BPA for CTEPH patients involves a complicated variety of indexes, including symptoms, biomarkers, echocardiographic results, and hemodynamic parameters.^[5, 6, 7, 8]^ The value of subjective outcomes, such as quality of life (QoL), as factors for evaluating the treatment effect of BPA in these patients has not been clearly addressed.

QoL is clinically assessed using a questionnaire that evaluates factors influencing the patient’s daily life. Thus, QoL measurement for patients with CTEPH provides an essential insight into the patients’ subjective perception of the impacts of CTEPH and BPA treatment. Previous studies have demonstrated an improvement in the QoL of patients with pulmonary arterial hypertension (PAH) following medical treatments and in CTEPH patients after BPA.^[9, 10, 11]^ Accordingly, we sought to investigate whether QoL measurement can be applied to assess the treatment effect of BPA in CTEPH patients, and if so, whether QoL score could serve as one of the treatment endpoints for BPA in these patients.

## Materials and methods

### Patients

The research related to human use has been complied with all the relevant national regulations, institutional policies, and in accordance with the tenets of the Helsinki Declaration, and has been approved by the Beijing Chaoyang Hospital Ethics Committee (2018-KE-144). CTEPH was diagnosed according to the International Guidelines of Pulmonary Hypertension (2015 ERS/ ESC Guidelines for Pulmonary Hypertension),^[12]^ in which CTEPH is defined by (1) mean pulmonary arterial pressure (mPAP) ≥25 mmHg and pulmonary arterial wedge pressure (PAWP) ≤15 mmHg confirmed by right heart catheterization (RHC), (2) chronic thrombosis confirmed by mismatched perfusion defects on ventilation/perfusion scanning or computed tomography angiography, and (3) at least 3 months of regular anticoagulation therapy. CTEPH patients were recruited if they (1) were confirmed inoperable by a multidisciplinary team, (2) had received at least four BPA sessions in our center, and (3) were aged ≥18 years. Patients were excluded if they were concomitant with type III or V pulmonary hypertension or had mental illness or could not complete a valid QoL questionnaire. The feasibility of pulmonary endarterectomy was determined by a multidisciplinary team, including physicians, radiologists, and surgeons.^[13]^

Informed consent was obtained from all individuals included in this study.

### Clinical data collection

The following demographic and clinical data were collected before the first and after each BPA session: age, gender, duration of CTEPH (from onset of symptoms to treatment), number of BPA sessions, symptoms, World Health Organization functional classification (WHO FC), 6-min walk distance (6MWD), laboratory examination, and echocardiography parameters. RHC parameters were also collected before the first BPA session. For QoL assessment, patients completed the RAND 36-item short-form (SF-36v2) questionnaire from the Medical Outcomes Study before and after every two BPA sessions.

RHC data were also collected 4–6 months after the last BPA session and considered as follow-up records. At the same time, the physical component summary (PCS) scores and the mental component summary (MCS) scores from the SF-36v2 for these patients were used as follow-up data.

### BPA procedure

BPA was performed by two skilled interventional radiologists. A conventional femoral venipuncture was made. A 6F JR- or JL-guided catheter (Launcher; Cordis, Miami Lakes, FL, USA) was introduced to the target vessel by a guidewire. A 2-mm balloon was used for the first vascular expansion. Then, the balloon was gradually inflated to a size no larger than the target vessel. If pulmonary artery injury occurred during the operation, the balloon was immediately deflated. If this was not practical, a gelatin sponge was used. Two oral anticoagulants, warfarin or rivaroxaban, were routinely used after BPA. For warfarin use, the international normalized ratio was maintained between 2.0 and 3.0.

The odd-numbered intervals between BPA sessions were no less than 2 weeks, and the even-numbered intervals were no less than 1 month. The number of BPA sessions, total number of dilated vessels (segmental level), and number of dilated vessels per session were recorded.

### Right heart catheterization

RHC was performed in all patients using a Philips monitoring system (Shenzhen Goldway Industrial Inc., Shenzhen, China) on the day of admission and after every two sessions of BPA to record hemodynamic factors. Briefly, a Swan-Ganz catheter was inserted via the jugular vein to assess the pressure in the right ventricle and pulmonary artery. The mPAP was recorded, and oximetry samples were obtained. The PAWP was calculated to exclude post-capillary pulmonary hypertension. The cardiac output (CO) was calculated using thermodilution, and the mean values from triplicate measurements are provided. The pulmonary vascular resistance (PVR) was calculated using the formula PVR = (mPAP – PAWP)/ CO. Pulmonary angiography was performed for bilateral pulmonary arteries at baseline.

### Assessment of QoL

The SF-36v2 (Chinese version) has been previously used by us to evaluate the QoL of CTEPH patients.^[14]^ In the present study, SF-36v2 data were collected at baseline and after every two BPA sessions. The patients were asked to complete the questionnaire using an online survey platform on a smartphone application called Questionnaire Star. For patients who did not have a smartphone, the questionnaire was administered by a doctor. The SF-36v2 score describes the overall health status of the patients in eight different domains—physical functioning (PF), bodily pain (BP), general health (GH), role-emotional (RE), role-physical (RP), social functioning (SF), vitality (VT), and mental health (MH).

The results of the questionnaire were transformed to a score of 0–100, and the scores obtained before and after BPA treatment were compared. The PCS and MCS scores were recorded at each assessment.

### Statistical analysis

All statistical analyses were performed using Statistical Package for the Social Sciences (SPSS) version 25 (SPSS Inc., Chicago, IL, USA). Data for non-normally distributed continuous variables are presented as the median and interquartile range. Data for continuous variables at baseline and after BPA were compared using the Wilcoxon’s signed-rank test. The categorical data, including gender, WHO FC, and general health condition, are presented as percentages and were compared using the Chi-squared test.

Receiver operating characteristic (ROC) curve analysis was performed to determine the PCS level that provided the best combination of sensitivity and specificity for predicting the WHO FC before and after BPA treatment. Patients were then divided into two groups according to the cutoff PCS score at baseline and after treatments, respectively. The Spearman correlation coefficient for rank data was used to estimate the association between hemodynamic status and PCS/MCS before and after BPA. Friedman’s test (Wilcoxon’s signed-rank test between the two groups with post hoc analysis) was used to compare the hemodynamic parameters from RHC, 6MWD, and clinical data between different stages. The level of statistical significance was set at *P* < 0.05.

## Results

### Demographic and clinical characteristics of patients at baseline

A total of 40 patients (11 males and 29 females) were enrolled in this study. The mean patient age was 58.5 ± 10.7 years, and the median duration of CTEPH from symptom onset to therapy was 5 (3, 9) years. All 40 patients (100%) complained of dyspnea, while chest congestion was reported by 37 patients (93%), edema of a lower extremity by 27 patients (68%), and transient syncope by eight patients (20%). Among all participants, 13 were classified as WHO FC II, 20 patients as WHO FC III, and seven patients as WHO FC IV. The baseline hemodynamic measurements included a mPAP of 50.7 ± 11.2 mmHg, PVR of 913.9 ± 338.2 dyn·s^−1^·m^−5^, and cardiac index (CI) of 2.27 ± 0.60 L·min^−1^·m^−2^. Before BPA treatment, 32 patients were receiving targeted drug therapy (Table 1).

**Table 1:** Characteristics of the patients with CTEPH

Parameters	Values (*n* = 40)
Age, years	58.5 ± 10.7
Male/female, *n*	11/29
Medical history, years	5 (3, 9)
Number of BPA sessions, *n*	4 (4, 4.75)
Total number of dilated vessels, *n*	22 (18.5, 26)
Symptoms	
Dyspnea, %	100
Chest congestion, %	93
Edema of lower extremity, %	68
Chest pain, %	13
Transient syncope, %	20
Hemoptysis, %	15
WHO FC, *n*	
I	0
II	13
III	20
IV	7
6MWD, m	354 (269, 433)
PaO_2_, mmHg	58.3 ± 8.3
SvO_2_, %	57.1 ± 9.3
mPAP, mmHg	50.7 ± 11.2
RAP, mmHg	8 (5, 11)
CO, L·min^−1^	3.83 ± 1.12
CI, L·min^−1^·m^−2^	2.27 ± 0.60
PVR, dyn·s^−1^·m^−5^	913.9 ± 338.2
Diuretics usage, *n*	38
Targeted drugs usage, *n*	32

CTEPH: chronic thromboembolic pulmonary hypertension; BPA: balloon pulmonary angioplasty; WHO FC: World Health Organization functional classification; 6MWD: 6-min walk distance; PaO_2_: oxygen arterial pressure of indoor air; SvO_2_: mixed venous oxygen saturation; mPAP: mean pulmonary arterial pressure; RAP: right arterial pressure; CO: cardiac output; CI: cardiac index; PVR: pulmonary vascular resistance.

Medical history: duration from the onset of symptoms to treatment.

### Changes in hemodynamics, clinical parameters, and QoL scores before and after BPA

A total of 176 BPA sessions were performed from January 12, 2017 to July 12, 2020. The median number of BPA sessions per patient was 4 (4, 4.75), and the median total number of dilated vessels per patient was 22 (18.5, 26) (Table 1). Guidewire-related pulmonary artery injury occurred in five cases, and all of the patients presented with cough. Only one patient presented with hemoptysis, which stopped after vessel inflation. No case of reperfusion pulmonary edema was observed.

**Figure 1:**
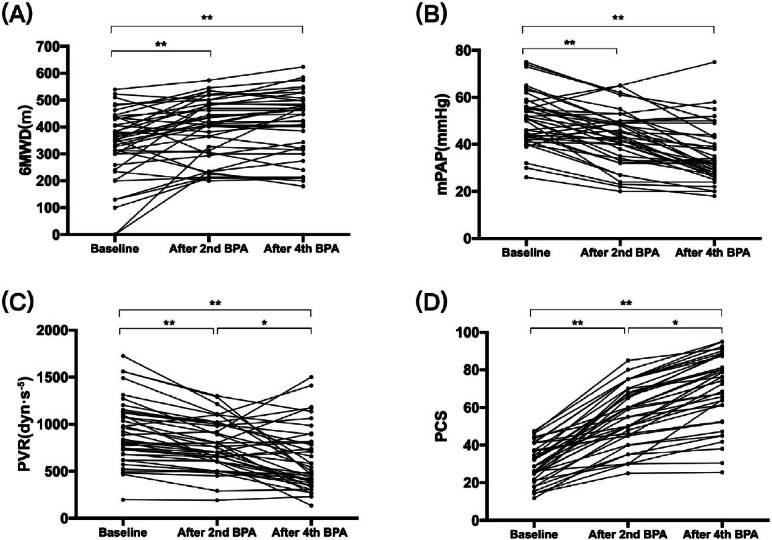
The change of (A) 6MWD, (B) mPAP, (C) PVR, (D) PCS along with BPA procedures. It shows that with the process of BPA, hemodynamics improved gradually. 6MWD: 6-min walk distance, mPAP: mean pulmonary arterial pressure, PVR: pulmonary vascular resistance, PCS: physical component summary, BPA: balloon pulmonary angioplasty. ^*^P < 0.05, ^**^P < 0.001.

Comparisons of clinical factors from before to after multiple BPA sessions (four to six procedures) are presented in Table 2 and Figure 1. After several procedures of BPA, the proportion of patients classified as WHO FC III/IV decreased from 67.5% to 30% (*P* < 0.001), and the median N-terminal of the prohormone brain natriuretic peptide (NT-proBNP) also decreased from 1139.5 (287.8, 3505.3) to 126.5 (72.8, 1077) pg/mL. Meanwhile, the oxygen arterial pressure of indoor air (PaO_2_) increased from 58.3 ± 8.3 to 65.5 ± 9.8 mmHg (*P* < 0.001), the mixed venous oxygen saturation (SvO_2_) increased from 57.1% ± 9.3% to 60.5% ± 8.0% (*P* = 0.042), and the 6MWD increased from 354 (269, 433) to 455 (322, 499) m (*P* < 0.001). With respect to hemodynamic parameters, the median mPAP and mean PVR decreased significantly from 51 (43, 56) to 33 (27, 43.8) mmHg (*P* < 0.001) and from 864 (680, 1128) to 520 (392, 808) dyn·s^−1^·m^−5^ (*P* < 0.001), respectively, while the heart rate reduced from 89 (74, 94) to 82 (72, 88) (*P* = 0.023). However, the improvement in CO was not statistically significant. Echocardiographic results showed an increase in tricuspid annular plane systolic excursion (TAPSE) from 14.0 ± 3.1 to 16.6 ± 3.9 mm (*P* < 0.001), a reduction in tricuspid regurgitation pressure gradient (TRPG) from 81.5 ± 24.3 to 63.6 ± 31.1 mmHg (*P* < 0.001), and a decrease in the right ventricular internal diameter/end-diastolic diameter of the left ventricle (RV/LV) ratio from 1.2 (1.0, 1.4) to 0.89 (0.74, 1.02) (*P* < 0.001).

**Table 2:** Changes in hemodynamics and clinical parameters before and after BPA

Parameters	*n*	Before BPA	After BPA	*P* value
NT-pro BNP, pg/mL	40	1139.5 (287.8, 3505.3)	126.5 (72.8, 1077)	<0.001
mPAP, mmHg	40	51 (43, 56)	33 (27, 43.8)	<0.001
PVR, dyn·s^−1^·m^−5^	40	864 (680, 1128)	520 (392, 808)	<0.001
RAP, mmHg	40	8 (5, 10.75)	5 (3, 9.5)	0.006
CO, L·min^−1^	40	3.55 (3.17, 4.41)	3.79 (3.54, 4.21)	0.528
CI, L·min^−1^·m^−2^	40	2.27 ± 0.60	2.37 ± 0.59	0.274
HR	40	89 (74, 94)	82 (72, 88)	0.023
SvO_2_, %	40	57.1 ± 9.3	60.5 ± 8.0	0.042
WHO FC, *n* (%)	40			
I or II	40	13	28	0.001
III or V	40	27	12	
6MWD, m	40	354 (269.3, 433.5)	455 (322.5, 499.3)	<0.001
PaO_2_, mmHg	40	58.3 ± 8.3	65.5 ± 9.8	<0.001
Echocardiography	40			
TAPSE, mm		14.0 ± 3.1	16.6 ± 3.9	<0.001
RV/LV		1.21 (1.02, 1.39)	0.89 (0.74, 1.02)	<0.001
TRPG, mmHg		81.5 ± 24.3	63.6 ± 31.1	<0.001
PCS	40	30.4 ± 9.9	69.7 ± 18.5	<0.001
MCS	40	46.4 ± 19.0	81.6 ± 13.2	<0.001

BPA: balloon pulmonary angioplasty; NT-proBNP: N-terminal of the prohormone brain natriuretic peptide; mPAP: mean pulmonary arterial pressure; PVR: pulmonary vascular resistance; RAP: right arterial pressure; CO: cardiac output; CI: cardiac index; HR: heart rate; SvO_2_: mixed venous oxygen saturation; WHO FC: World Health Organization functional classification; 6MWD: 6-min walk distance; PaO_2_: oxygen arterial pressure of indoor air; TAPSE: tricuspid annular plane systolic excursion; RV/LV: right ventricular internal diameter/end-diastolic diameter of the left ventricle; TRPG: tricuspid regurgitation pressure gradient; PCS: physical component summary; MCS: mental component summary.

**Figure 2:**
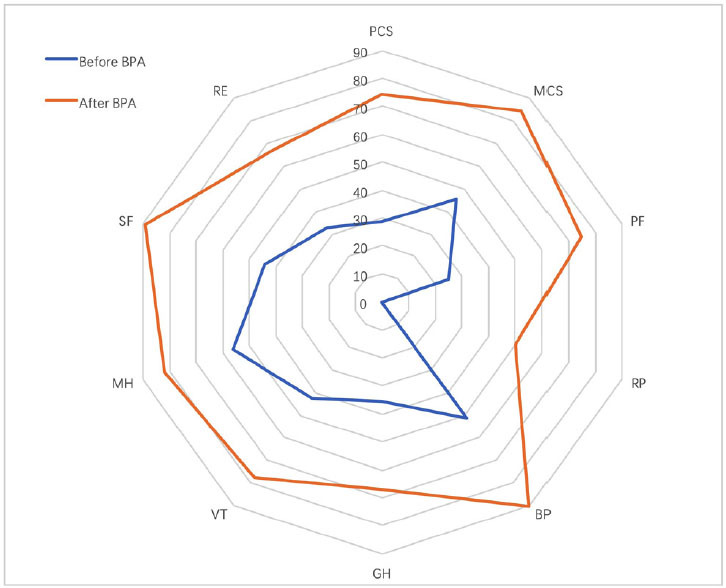
The changes in QoL before and after BPA. QoL: quality of life, BPA: balloon pulmonary angioplasty, PF: physical functioning, BP: bodily pain, GH: general health, RE: role-emotional, RP: role-physical, SF: social functioning, VT: vitality, MH: mental health, PCS: physical component summary, MCS: mental component summary.

Improvements in QoL scores also were seen after BPA, with the PCS and MCS scores increasing significantly from 30.4 ± 9.9 to 69.7 ± 18.5 and from 46.4 ± 19.0 to 81.6 ± 13.2 (both *P* < 0.001), respectively. The PF, RP, BP, GH, VT, SF, RE, and MH scores also increased significantly (Figure 2).

### Comparison of clinical characteristics according to the PCS score at baseline and after therapy

ROC analysis showed that 35 was the cutoff value before treatment, and patients who had a PCS score of ≥35 at baseline had longer 6MWD (429 *vs*. 327 m, *P* = 0.03), lower NT-proBNP level (265.5 *vs*. 2362 pg/mL, *P* = 0.002), lower PVR (716 *vs*. 1009 dyn·s^−1^·m^−5^, *P* = 0.009), lower right arterial pressure (RAP; 5 *vs*. 9 mmHg, *P* = 0.02), lower right ventricular diameter (RVD; 43 *vs*. 50 mm, *P* = 0.019), lower RV/LV ratio (0.97 *vs*. 1.31, *P* = 0.005), higher TAPSE (15.9 *vs*. 13 mm, *P* = 0.005), greater CO (4.4 *vs*. 3.6 L/min, *P* = 0.028), and higher CI (2.5 *vs*. 2.1 L min^−1^ m^−2^, *P* = 0.039) (Table 3).

**Table 3:** Baseline cardiac and hemodynamic differences according to PCS classification

Parameters	PCS ≥ 35 *n* = 13	PCS ≤ 35*n* = 27	*P* value
6MWD, m	429 (350, 471.5)	327 (240, 384)	0.030
NT-proBNP, pg/mL	265.5 (208.75, 706.25)	2362 (738.3, 3728.5)	0.002
RVD, mm	43.1 ± 7.4	50.0 ± 8.4	0.019
TAPSE, mm	15.9 ± 3.5	13.0 ± 2.5	0.005
TRPG, mmHg	78.5±20.5	83.0 ± 26.1	0.453
RV/LV	0.97 (0.89, 1.20)	1.31 (1.11, 1.41)	0.005
RAP, mmHg	5 (3.5, 9)	9 (6, 11)	0.020
mPAP, mmHg	45 (43, 55.5)	52 (42, 59)	0.333
CO, L·min^−1^	4.4 ± 0.9	3.6 ± 1.1	0.028
CI, L·min^−1^·m^−2^	2.5 ± 0.5	2.1 ± 0.6	0.039
PVR, dyn·s^−1^·m^−5^	716.3 ± 192.3	1009 ± 354.4	0.009

PCS: physical component summary; 6MWD: 6-min walk distance; NT-proBNP: N-terminal of the prohormone brain natriuretic peptide; RVD: right ventricular diameter; TAPSE: tricuspid annular plane systolic excursion; TRPG: tricuspid regurgitation pressure gradient; RV/LV: right ventricular internal diameter/end-diastolic diameter of the left ventricle; RAP: right arterial pressure; mPAP: mean pulmonary arterial pressure; CO: cardiac output; CI: cardiac index; PVR: pulmonary vascular resistance.

In addition, a PCS score of 46 was set as the cutoff value after therapy according to ROC analysis (Appendix Figure). Patients who had a PCS of >46 after BPA had better 6MWD (463 *vs*. 287 m, *P* = 0.047), CO (3.83 *vs*. 3.28 L/min, *P* = 0.031), CI (2.47 *vs*. 1.71 L min^−1^ m^−2^, *P* = 0.006), and PVR (594.36 *vs*. 910.00 dyn·s^−1^·m^−5^, *P* = 0.048) (Table 4).

**Table 4:** Cardiac and hemodynamic differences according to PCS classification after BPA

Parameters	PCS ≥ 46 *n* = 34	PCS ≤ 46*n* = 6	*P* value
6MWD, m	463.0 (374.5, 501.8)	287.0 (233.3, 362.0)	0.047
NT-proBNP, pg/mL	124.5 (69.8, 1039)	632.5 (87.8, 2568.8)	0.272
RVD, mm	39.53 ± 8.91	45.50 ± 11.88	0.158
TAPSE, mm	16.80 ± 4.00	15.43 ± 3.21	0.433
TRPG, mmHg	61.77 ± 19.00	64.24 ± 34.86	0.872
RV/LV	0.90 ± 0.24	1.08 ± 0.41	0.142
RAP, mmHg	5 (3, 9)	9 (3, 11)	0.562
mPAP, mmHg	33 (27, 43)	42 (23, 59)	0.649
CO, L·min^−1^	3.83 (3.66, 4.24)	3.28 (2.18, 2.68)	0.031
CI, L·min^−1^·m^−2^	2.47 ± 0.55	1.71 ± 0.40	0.006
PVR, dyn·s^−1^m^−5^	594.36 ± 303.00	910.00 ± 444.50	0.048

PCS: physical component summary; 6MWD: 6-min walk distance; NT-proBNP: N-terminal of the prohormone brain natriuretic peptide; RVD: right ventricular diameter; TAPSE: tricuspid annular plane systolic excursion; TRPG: tricuspid regurgitation pressure gradient; RV/LV: right ventricular internal diameter/end-diastolic diameter of the left ventricle; RAP: right arterial pressure; mPAP: mean pulmonary arterial pressure; CO: cardiac output; CI: cardiac index; PVR: pulmonary vascular resistance.

### Correlation between QoL improvement and clinical or hemodynamic improvements after BPA

**Figure 3:**
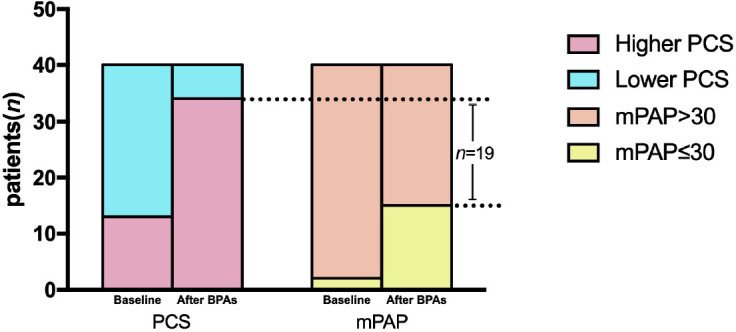
The different changes of PCS and mPAP before and after BPAs. It demonstrated that after four sessions of BPA, 19 patients (55.9%) who had a better PCS (PCS ≥ 46) after treatment still did not achieve the goal of a mPAP ≤30 mmHg. PCS: physical component summary, mPAP: mean pulmonary arterial pressure, BPA: balloon pulmonary angioplasty.

There was no linear dependence between the change in QoL and clinical or hemodynamic improvement after BPA for these patients. Besides, 19 patients (55.9%) who had already got a better PCS (>46) after treatment did not achieve the goal of mPAP <P30 mmHg after BPA (Figure 3). For patients who underwent more than four sessions of BPA, the 6MWD, mPAP, and PVR improved gradually with each additional BPA session; however, the PCS score improved more rapidly than the other parameters.

### Changes in hemodynamics and PCS score during follow-up

**Figure 4:**
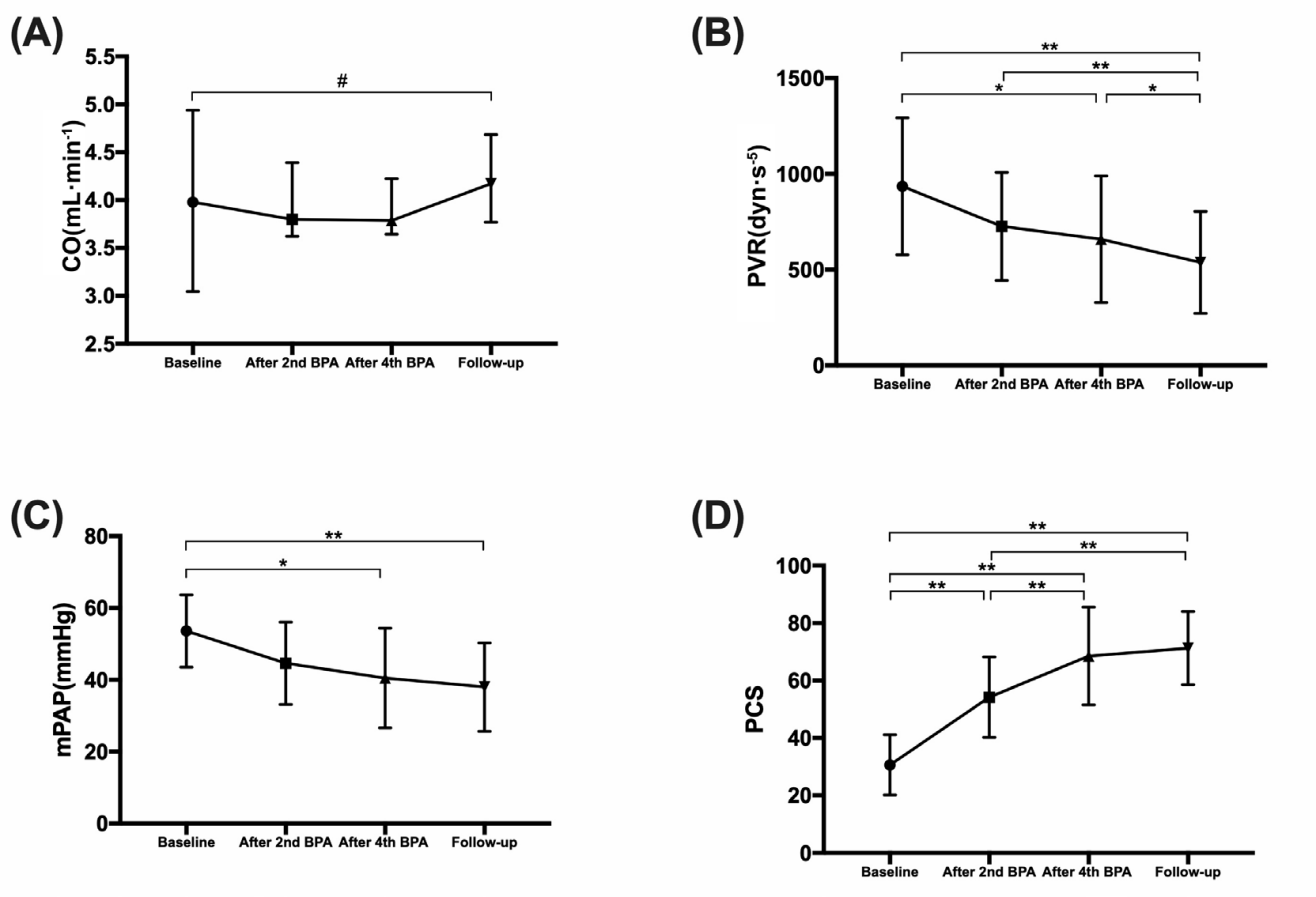
The trend of (A) CO, (B) PVR, (C) mPAP, and (D) PCS from baseline to follow-up of 20 patients who had 4–6 months of follow-up. It demonstrated that PVR still improved in the follow-up, while PCS tended to be stable. PCS: physical component summary, CO: cardiac output, PVR: pulmonary vascular resistance, mPAP: mean pulmonary arterial pressure. ^#^*P* > 0.05, ^*^P < 0.05, ^**^P < 0.001.

During a follow-up period of 4–6 months after the last BPA procedure, 20 patients underwent RHC. For these 20 patients, the mPAP increased slightly from 35 (31, 48) to 38 (27, 45) mmHg between the last BPA and the last follow-up, while the RAP changed from 5 (4, 7) to 4 (3, 5) mmHg and the CO increased from 3.8 (3.6, 4.2) to 4.2 (3.8, 4.7) L·min^−1^. However, these differences were not significant. Additionally, the PCS score of these patients did not improve significantly from the last BPA procedure to the final follow-up (68.5 *vs*. 71.3, *P* = 0.287). PVR was the only parameter to show significant improvement during follow-up, with a decrease from 659.6 ± 331.5 to 538.3 ± 266.4 dyn·s^−1^·m^−5^ (*P* = 0.045; Figure 4).

## Discussion

To the best of our knowledge, the treatment endpoint of BPA remains a subject of debate and this is the first study to investigate the role of QoL in assessing the treatment effect of BPA in patients with inoperable CTEPH. The main findings of this study were as follows: (1) significant improvements in hemodynamic parameters, clinical factors, and QoL scores were observed after more than four sessions of BPA; (2) PCS score can be used to predict activity endurance and hemodynamic status both before and after BPA; and (3) PCS cannot be considered a treatment endpoint for patients with CTEPH.

BPA can significantly improve hemodynamic parameters and QoL among CTEPH patients, as confirmed by the present and previous studies.^[9, 15, 16, 17]^ However, the appropriate time to stop BPA remains unknown. An established treatment goal of BPA is to improve the hemodynamic kinetics and oxygen consumption in CTEPH patients.^[6,18,19]^ Currently, a mPAP ≤30 mmHg and SpO_2_>95% are considered potential indications for BPA.^[5, 20]^ However, in real-world practice, clinical cure among these patients is rare due to residual lesions and financial concerns.^[6, 21]^ It remains to be determined whether BPA can be stopped once patients show a significant improvement in exercise ability as well as what role can QoL play in planning the BPA treatment strategy. In the present study, 19 patients (55.9%), whose PCS score was already over 46 after BPA, had a mPAP higher than 30 mmHg. We also found that the change in QoL and that of clinical or hemodynamic data were not parallel during treatment. These may imply that the PCS score improved faster than mPAP, so that PCS insufficiently predicted when BPA could be stopped and for patients who feel subjectively better after initial BPA, the interventional procedures should be continued. Collectively, although PSC scores of 35 and 46 can be used as the cutoff values to predict the activity function of patients with CTEPH, they cannot be considered a treatment endpoint.

Previous studies have shown that during follow-up, improvements in hemodynamic parameters and vascular morphology continued after the last BPA session.^[22, 23]^ The results of the present study also support this: during the median 4–6 months of follow-up, the hemodynamic parameters showed continued improvement. However, the PCS scores remained stable during follow-up, indicating that there is a time lag between improvement in hemodynamic parameters and QoL. Therefore, it is essential to evaluate the effect of BPA sequentially, even though QoL has shown obvious improvement. To sum up, both in terms of the degree of improvement and the time of improvement, the PCS score cannot be taken as the major factor for judging the cessation of BPA.

Several nonspecific and specific QoL tools are available for PAH and CTEPH patients.^[11, 24]^ We chose the SF-36 for the assessment of QoL in this study because it can evaluate the effects of medical therapy, surgery, and exercise training on eight health domains of patients and has been widely used in patients with PAH and CTEPH.^[25, 26, 27, 28, 29]^ The PCS score offers a comprehensive assessment of physical endurance, and normal people also have a normal range of the PCS score. However, for CTEPH patients, the appropriate PCS cutoff value remains to be confirmed. In the present study, we showed PCS scores of 35 and 46 as the cutoff values for differentiating activity endurance and cardiac function before and after BPA. Similarly, a previous study reported a PCS threshold value of 32 and 38 for PAH patients before and after treatment, respectively.^[30]^ More improvement in PCS was observed in our patients than in PAH patients after treatment, suggesting that BPA may increase the PCS score in CTEPH patients more prominently than the drug does for PAH patients.

In the present study, CO tended to increase within the process of BPA procedures, but without a significant difference. We deduced that this was because an inadequate number of BPA sessions had been completed. This phenomenon was also found in a previous study, which showed that nonparallel improvement of CO and other hemodynamic parameters may be due to an inadequate number of BPA sessions and suggested that more BPA sessions were needed.^[16]^ In addition, patients who were enrolled in this study had relatively slight decreased CO, which cannot be improved dramatically. On the other hand, we found that the RV area, RV/LV, RAP, and TAPSE on echocardiography were improved significantly after treatment, which likely means that the improvement of right heart structure detected on echocardiography is earlier than that of data detected by RHC and more studies should be done in this area in the future.

The present study has some limitations that must be mentioned. Firstly, the sample size was relatively small. In addition, due to the retrospective study design, not all patients’ follow-up data were collected. Further multicenter, prospective studies focusing on QoL and BPA are needed.

## References

[1] Ogawa A, Matsubara H. (2015). Balloon Pulmonary Angioplasty: A Treatment Option for Inoperable Patients with Chronic Thromboembolic Pulmonary Hypertension. Front Cardiovasc Med.

[2] Andreassen AK, Ragnarsson A, Gude E, Geiran O, Andersen R. (2013). Balloon pulmonary angioplasty in patients with inoperable chronic thromboembolic pulmonary hypertension. Heart.

[3] Delcroix M, Torbicki A, Gopalan D, Sitbon O, Klok FA, Lang I (2021). ERS Statement on Chronic Thromboembolic Pulmonary Hypertension. Eur Respir J.

[4] Kim NH, Delcroix M, Jais X, Madani MM, Matsubara H, Mayer E (2019). Chronic thromboembolic pulmonary hypertension. Eur Respir J.

[5] Kataoka M, Inami T, Kawakami T, Fukuda K, Satoh T. (2019). Balloon Pulmonary Angioplasty (Percutaneous Transluminal Pulmonary Angioplasty) for Chronic Thromboembolic Pulmonary Hypertension: A Japanese Perspective. JACC Cardiovascular interventions.

[6] Aoki T, Sugimura K, Tatebe S, Miura M, Yamamoto S, Yaoita N (2017). Comprehensive evaluation of the effectiveness and safety of balloon pulmonary angioplasty for inoperable chronic thrombo-embolic pulmonary hypertension: long-term effects and procedure-related complications. Eur Heart J.

[7] Roller FC, Kriechbaum S, Breithecker A, Liebetrau C, Haas M, Schneider C (2019). Correlation of native T1 mapping with right ventricular function and pulmonary haemodynamics in patients with chronic thromboembolic pulmonary hypertension before and after balloon pulmonary angioplasty. Eur Radiol.

[8] Kriechbaum SD, Wiedenroth CB, Peters K, Barde MA, Ajnwojner R, Wolter JS (2020). Galectin-3, GDF-15, and sST2 for the assessment of disease severity and therapy response in patients suffering from inoperable chronic thromboembolic pulmonary hypertension. Biomarkers.

[9] Darocha S, Pietura R, Pietrasik A, Norwa J, Dobosiewicz A, Piłka M (2017). Improvement in Quality of Life and Hemodynamics in Chronic Thromboembolic Pulmonary Hypertension Treated With Balloon Pulmonary Angioplasty. Circ J.

[10] Arvanitaki A, Mouratoglou SA, Evangeliou A, Grosomanidis V, Hadjimiltiades S, Skoura L (2020). Quality of Life is Related to Haemodynamics in Precapillary Pulmonary Hypertension. Heart Lung Circ.

[11] Cenedese E, Speich R, Dorschner L, Ulrich S, Maggiorini M, Jenni R (2006). Measurement of quality of life in pulmonary hypertension and its significance. Eur Respir J.

[12] Galie N, Humbert M, Vachiery JL, Gibbs S, Lang I, Torbicki A (2016). 2015 ESC/ERS Guidelines for the diagnosis and treatment of pulmonary hypertension: The Joint Task Force for the Diagnosis and Treatment of Pulmonary Hypertension of the European Society of Cardiology (ESC) and the European Respiratory Society (ERS): Endorsed by: Association for European Paediatric and Congenital Cardiology (AEPC), International Society for Heart and Lung Transplantation (ISHLT). Eur Heart J.

[13] Jenkins D, Madani M, Fadel E, D’Armini AM, Mayer E. (2017). Pulmonary endarterectomy in the management of chronic thromboembolic pulmonary hypertension. Eur Respir Rev.

[14] Zhou X, Shi H, Yang Y, Zhang Z, Zhai Z, Wang C. (2020). Anxiety and depression in patients with pulmonary arterial hypertension and chronic thromboembolic pulmonary hypertension: Results from a Chinese survey. Exp Ther Med.

[15] Jansa P, Heller S, Svoboda M, Pad’our M, Ambrož D, Dytrych V (2020). Balloon Pulmonary Angioplasty in Patients with Chronic Thromboembolic Pulmonary Hypertension: Impact on Clinical and Hemodynamic Parameters, Quality of Life and Risk Profile. J Clin Med.

[16] Kwon W, Yang JH, Park TK, Chang SA, Jung DS, Cho YS (2018). Impact of Balloon Pulmonary Angioplasty on Hemodynamics and Clinical Outcomes in Patients with Chronic Thromboembolic Pulmonary Hypertension: the Initial Korean Experience. J Korean Med Sci.

[17] Hoole SP, Coghlan JG, Cannon JE, Taboada D, Toshner M, Sheares K (2020). Balloon pulmonary angioplasty for inoperable chronic thromboembolic pulmonary hypertension: the UK experience. Open Heart.

[18] Ogawa A, Satoh T, Fukuda T, Sugimura K, Fukumoto Y, Emoto N (2017). Balloon Pulmonary Angioplasty for Chronic Thromboembolic Pulmonary Hypertension: Results of a Multicenter Registry. Circ Cardiovasc Qual Outcomes.

[19] Wang W, Wen L, Song Z, Shi W, Wang K, Huang W. (2019). Balloon pulmonary angioplasty vs riociguat in patients with inoperable chronic thromboembolic pulmonary hypertension: A systematic review and meta-analysis. Clin Cardiol.

[20] Tsuji A, Ogo T, Ueda J, Fukui S, Morita Y, Fukuda T (2017). Predictors of residual pulmonary hypertension after balloon pulmonary angioplasty in patients with chronic thromboembolic pulmonary hypertension. Int J Cardiol.

[21] Sugimura K, Fukumoto Y, Satoh K, Nochioka K, Miura Y, Aoki T (2012). Percutaneous transluminal pulmonary angioplasty markedly improves pulmonary hemodynamics and long-term prognosis in patients with chronic thromboembolic pulmonary hypertension. Circ J.

[22] Shimokawahara H, Nagayoshi S, Ogawa A, Matsubara H. (2021). Continual Improvement in Pressure Gradient at the Lesion After Balloon Pulmonary Angioplasty for Chronic Thromboembolic Pulmonary Hypertension. Can J Cardiol.

[23] Inami T, Kataoka M, Yanagisawa R, Ishiguro H, Shimura N, Fukuda K (2016). Long-term outcomes after percutaneous transluminal pulmonary angioplasty for chronic thromboembolic Pulmonary Hypertension. Circulation.

[24] Minatsuki S, Kodera S, Kiyosue A, Saito A, Maki H, Hatano M (2020). Balloon pulmonary angioplasty improves quality of life in Japanese patients with chronic thromboembolic pulmonary hypertension. J Cardiol.

[25] Mathai SC, Ghofrani H-A, Mayer E, Pepke-Zaba J, Nikkho S, Simonneau G. (2016). Quality of life in patients with chronic thromboembolic pulmonary hypertension. Eur Respir J.

[26] Mathai SC, Suber T, Khair RM, Kolb TM, Damico RL, Hassoun PM. (2016). Health-related Quality of Life and Survival in Pulmonary Arterial Hypertension. Ann Am Thorac Soc.

[27] Kamenskaya O, Klinkova A, Loginova I, Chernyavskiy A, Lomivorotov VV, Karaskov A. (2017). Factors affecting the quality of life before and after surgery in patients with chronic thromboembolic pulmonary hypertension. Qual Life Res.

[28] Roman A, Barbera JA, Castillo MJ, Muñoz R, Escribano P. (2013). Health-Related Quality of Life in a National Cohort of Patients With Pulmonary Arterial Hypertension or Chronic Thromboembolic Pulmonary Hypertension. Arch Bronconeumol.

[29] Yoshimi S, Tanabe N, Masuda M, Sakao S, Uruma T, Shimizu H (2008). Survival and quality of life for patients with peripheral type chronic thromboembolic pulmonary hypertension. Circ J.

[30] Fernandes CJ, Martins BC, Jardim CV, Ciconelli RM, Morinaga LK, Breda AP (2014). Quality of life as a prognostic marker in pulmonary arterial hypertension. Health Qual Life Outcomes.

